# High‐risk HPV positivity is a long‐term risk factor for recurrence after cervical excision procedure in women living with HIV

**DOI:** 10.1002/ijgo.13674

**Published:** 2021-06-10

**Authors:** Alberto Agarossi, Giovanni Delli Carpini, Francesco Sopracordevole, Matteo Serri, Luca Giannella, Barbara Gardella, Marta Maestri, Anna Del Fabro, Matilde Sansone, Maria Grazia Fallani, Annalisa Pieralli, Maria Michela Fasolo, Cristina Mazzali, Andrea Ciavattini

**Affiliations:** ^1^ Department of Obstetrics and Gynecology L. Sacco Hospital ASST‐Fatebenefratelli‐Sacco University of Milan Milan Italy; ^2^ Woman’s Health Sciences Department Gynecologic Section Università Politecnica delle Marche Ancona Italy; ^3^ Gynecological Oncology Unit Centro di Riferimento Oncologico – National Cancer Institute Aviano Italy; ^4^ Department of Obstetrics and Gynecology University of Pavia Fondazione IRCCS Policlinico Pavia Italy; ^5^ Regional Reference Center for AIDS and Infectious Diseases in Obstetrics and Gynecology AOU Federico II Naples Italy; ^6^ Maternal and Child Department AOUC Florence Firenze FI Italy; ^7^ Division Department of Infectious Diseases ASST Fatebenefratelli Sacco University Hospital Milan Italy; ^8^ Presidio Regionale HTA dei Dispositivi Medici ASST Grande Ospedale Metropolitano Niguarda Milan Italy

**Keywords:** cervical excisional procedure, cervical intraepithelial neoplasia, conization, HIV, recurrence

## Abstract

**Objective:**

To evaluate the risk factors for recurrence of high‐grade disease after cervical excision in women living with HIV (WLWH), with a specific interest in the role of high‐risk (HR‐) HPV positivity.

**Methods:**

Multicentric retrospective study conducted on WLWH who underwent cervical excision between January 1987 and June 2017 in six Italian institutions. The rate of high‐grade recurrence was determined. Risk factors for recurrence and HR‐HPV positivity were determined with the Log‐rank test and Cox proportional hazards regression models.

**Results:**

A total of 271 WLWH were included in the final analysis. A high‐grade recurrence was found in 58 (21.4%) patients. Age 41 years or more at inclusion and HR‐HPV positivity during follow up were independently associated with a higher risk of disease recurrence with relative risks of 4.15 (95% confidence interval [CI] 2.01–8.58, *P* < 0.001) and 5.18 (95% CI 2.12–12.67, *P* < 0.01), respectively. Age 41 years or more (relative risk 1.75, 95% CI 1.01–3.04, *P* = 0.047) resulted as a risk factor for HR‐HPV positivity during follow up.

**Conclusion:**

HR‐HPV positivity is a risk factor for recurrence after cervical excision in WLWH. Women older than 41 years may benefit from a long‐term yearly follow up. Future studies regarding HPV vaccination after treatment in WLWH may be useful, considering the protective role of the higher probability of HPV negativity in vaccinated women.

## Synopsis

High‐risk‐HPV positivity during long‐term follow up is a risk factor for high‐grade disease recurrence after cervical excisional treatment in women living with HIV.

## 1 INTRODUCTION

Human papillomavirus (HPV) infection is the most common sexually transmitted infection, with over 300 million infected women globally. Risk factors for acquiring HPV infection include multiple sexual partners, immunodeficiency status, early sexual debut, parity, use of contraceptives, and smoking.^1^ Compared with the general female population, women living with HIV (WLWH) have a higher prevalence of high‐risk HPV (HR‐HPV) infection, HR‐HPV persistent infection, higher risk of cervical intraepithelial neoplasia (CIN) lesions, and higher incidence of cervical cancer.^2^, ^3^ The presence of HIV and related immunosuppression significantly affects the natural history of HR‐HPV infection, increasing the susceptibility to acquiring HR‐HPV, reducing the rate of clearance, and favoring both the reactivation of latent infection and the persistence of HR‐HPV infection.^2^, ^3^ Severe immunosuppression, as reflected by low CD4^+^ T‐cell count or increased HIV loads, has been consistently associated with the risk of HR‐HPV infection itself and with the risk of preinvasive and invasive cervical lesions.^3^ Highly active antiretroviral therapy (HAART), together with the associated immune reconstitution, seems to improve the control of HR‐HPV infection and disease progression.^3^

To date, because primary prevention with HPV vaccination in WLWH, although promising, still does not have strong scientific validation and further studies are undoubtedly necessary,^4^, ^5^ cervical cancer prevention in WLWH is mainly based on secondary prevention, with early diagnosis and treatment of high‐grade CIN (CIN 2/3). Surgical cervical excision (i.e. loop electrosurgical excision procedure, laser conization, or cold‐knife conization) is the recommended procedure in the case of CIN 2/3.^6^ Although the surgical treatment of cervical cancer precursors is highly effective in immunocompetent women, with eradication in about 90% of cases, a lower efficacy has been reported in WLWH, in whom high rates of persistent and recurrent disease have been found in several studies.^7^ For this reason, a long‐term follow up is necessary for WLWH subjected to cervical excision for intraepithelial neoplasia.^8^ However, there is currently no consensus as to what the follow‐up method after cervical excision in WLWH should be. Available guidelines contain recommendations ranging from the same methods of follow up as HIV‐negative women to annual follow up, particularly in the case of severe immunodeficiency.^6^, ^9^, ^10^

Evidence suggests that several factors may be associated with increased rates of persistent and recurrent disease after treatment, including smoking, positivity of cone margins, persistent HR‐HPV infection, and immunosuppression.^11^ Persistent HR‐HPV infection is gaining increasing importance in the evaluation of the recurrence risk after treatment in HIV‐negative women,^12^ but evidence in this regard is scarce for WLWH. Knowing the impact of HPV infection on the risk of recurrence after treatment might also be the basis for future studies on the role of HPV vaccination after treatment in WLWH.

The aim of the present study was, therefore, to evaluate the risk factors for recurrence of high‐grade lesions after cervical excision in WLWH, with a specific interest in the role of HR‐HPV positivity during follow‐up.

## 2 MATERIALS AND METHODS

This was a multicentric, retrospective, cohort study that involved six Italian institutions. All WLWH who underwent cervical excision between January 1987 and June 2017 in the six institutions were retrospectively identified by searching the clinical databases and included in the present study.

The cervical treatment was performed because of high‐grade squamous intraepithelial lesions (HSIL, CIN2/3) or persistent (>2 years) low‐grade squamous intraepithelial lesions (LSIL, CIN1) diagnosis. Only women with a definitive diagnosis of high‐grade intraepithelial lesion (HSIL, CIN2/3) at the cone specimen were included in the final analysis. Women who reported previous treatment for cervical pathology were excluded. Likewise, patients with missing data and with a diagnosis of cancer on the first visit or after conization were not included in the analysis.

We collected pertinent sociodemographic and clinical data such as age, menopausal status, age at first sexual intercourse (≤16 years or ≥17 years), number of sexual partners (up to five or six or more), tobacco use, parity (nulliparous or parous), and reported route of transmission of HIV infection. Moreover, we collected data regarding the referral cytology, the colposcopic examination at inclusion, the histopathologic findings on pre‐operative biopsy, the final histopathologic diagnosis at the cone specimen, and the HR‐HPV status before cervical treatment and during follow up. Abnormalities on referral cervical cytology were classified according to the most recent Bethesda system terminology.^13^ Cytology examinations performed before the introduction of the most recent Bethesda system terminology were revised accordingly by the pathologists of each institution involved in the study. The colposcopic examinations were recorded according to the 2011 revised colposcopic terminology of the International Federation for Cervical Pathology and Colposcopy (IFCPC).^14^ The colposcopies performed before the introduction of the 2011 IFCPC nomenclature were revised accordingly by the gynecologists of each institution, through the revision of colposcopic charts. Histopathologic results were reported according to Lower Anogenital Squamous Terminology as LSIL (CIN1), HSIL (CIN2), HSIL (CIN3), or HSIL (CIN2/3).^15^ Cervical excisions were performed by experienced colposcopists (in practice for more than 10 years) working in each of the included institutions.

We also collected CD4^+^ T‐cell counts at inclusion and during follow up (at recurrence or the last negative follow up). CD4^+^ T‐lymphocyte count was recorded according to the Centers for Disease Control revised Surveillance Case Definition for HIV infection.^16^ According to the WHO immunologic classification for established HIV infection,^17^ we defined the following classes of HIV‐related immunodeficiency: none or not significant for CD4^+^ T‐cell count of 500 cells/µl or more, mild for a CD4^+^ T‐cell count of 350–499 cells/µl, advanced for a CD4^+^ T‐cell count of 200–349 cells/µl, and severe for a CD4^+^ T‐cell count below 200 cells/µl.

The use and type of antiretroviral therapy (ART) were recorded on the basis of the medications listed in the patients’ chart. HAART was defined according to the contemporary definition of two nucleoside reverse‐transcriptase inhibitors and at least one of the following: a protease inhibitor, a non‐nucleoside reverse‐transcriptase inhibitor, or an additional nucleoside reverse‐transcriptase inhibitors. All other types of ART used before 1996 were defined as pre‐HAART.

The post‐treatment follow‐up strategy was the following: first control with cervical cytology and colposcopy at 6 months from the procedure; after that, these patients underwent cervical cytology and colposcopy every 6 months until 24 months from treatment. After that, women underwent cervical cytology every 12 months with colposcopy in the case of abnormalities at cervical cytology. HR‐HPV testing was also added after 2002, when available, at the time of treatment and at yearly intervals. Biopsies were prompted by abnormal cytology or the presence of an abnormal transformation zone at colposcopy.

Disease recurrence was defined as a histopathologic diagnosis of high‐grade lesions (HSIL, CIN2/3) or worse (HSIL+) during follow up. These women were followed from the date of first follow up after treatment until the date of recurrence or until the date of the last registered follow up if no recurrence took place.

The primary outcome of the study was the rate of high‐grade recurrence or cancer (HSIL+) among WLWH.

All continuous variables were tested for normality with the D’Agostino‐Pearson test. Normally distributed variables were expressed as mean ±standard deviation, while skewed variables were reported as median and interquartile range (IQR). Categorical variables were reported as numbers and percentages. A Kaplan‐Meier analysis at 180 months of follow up for the whole population was performed to evaluate the probability of disease recurrence.

All sociodemographic, clinical, and HIV‐specific variables were evaluated as risk factors for disease recurrence with the log‐rank test. Factors that were associated with recurrence were considered as covariates in a subsequent Cox proportional‐hazards regression model. Risk factors for HPV‐positivity during follow up were evaluated with the same methodology. A *P* value <0.05 was considered statistically significant. Patients with missing data were excluded and a complete case analysis was performed. Calculations were done using IBM SPSS version 27.0 (IBM Corp., Armonk, NY, USA). As this was a retrospective analysis of routinely collected data, there was no need for ethical approval. Patients signed informed consent for data collection and procedure at the time of intervention.

## 3 RESULTS

During the study period, 345 WLWH underwent cervical excision in the six institutions involved. Thirty‐four women with incomplete data were excluded, leaving 311 for analysis. The majority of patients (281/311, 90.4%) were white, with a mean age ±standard deviation at inclusion of 35.5 ± 7.9 years. Fourteen (4.5%) women were in menopause at the time of treatment. The reported age at first intercourse was 16 years or less in 150 (48.2%) women, and 148 (47.6%) women reported a having five or fewer partners. Tobacco use was reported by 209 (67.2%) women. One hundred and sixty (51.5%) women were nulliparous at inclusion. Regarding HIV‐related characteristics, 171 (55.0%) had been infected by HIV via heterosexual contact. The median (IQR) CD4^+^ T‐cell count at inclusion was 365 (240–500) cells/µl. According to the WHO immunologic classification, 67 (21.5%) of these women presented a severe immunodeficiency, 66 (21.2%) an advanced immunodeficiency, 86 (27.7%) a mild immunodeficiency, and 92 (29.6%) a not significant immunodeficiency. At the time of treatment, 73 (23.5%) women were not receiving ART, 81 (26.0%) were receiving mono‐ or dual‐nucleoside reverse‐transcriptase inhibitors (pre‐HAART) therapy, and 157 (50.5%) were receiving HAART.

In the 165 women in whom an HR‐HPV test was performed at inclusion, the result was positive in 148 (89.7%). The preoperative diagnosis was HSIL (CIN2/3) at punch‐directed biopsy in 246 (79.1%) patients, high‐grade cervical cytology (HSIL or atypical squamous cells, cannot rule out high‐grade squamous intraepithelial lesion) in 7 (2.3%), persistent LSIL (CIN1) in 53 (17.0%), and persistent low‐grade cervical cytology (LSIL or atypical squamous cells of undetermined significance) in the remaining 5 (1.6%) patients.

At the preoperative colposcopy, the squamocolumnar junction was visible in 206 (66.2%) patients, and major colposcopic changes or signs of suspected invasion were observed in 167 (53.7%).

Treatment was performed by loop electrical excision procedure in 255 (82.0%) patients, by CO_2_‐laser in 40 (12.9%), and by cold knife in the remaining 16 (5.1%). At the histopathologic analysis of the cone specimens, the cone result was negative in 3 (1.0%) cases, LSIL (CIN1) in 23 (7.4%) patients, HSIL (CIN2) in 137 (44.1%), HSIL (CIN3) in 140 (45.0%), and invasive cancer in eight (2.5%) patients. The endocervical margin was negative in 272 (87.5%) patients.

**FIGURE 1:**
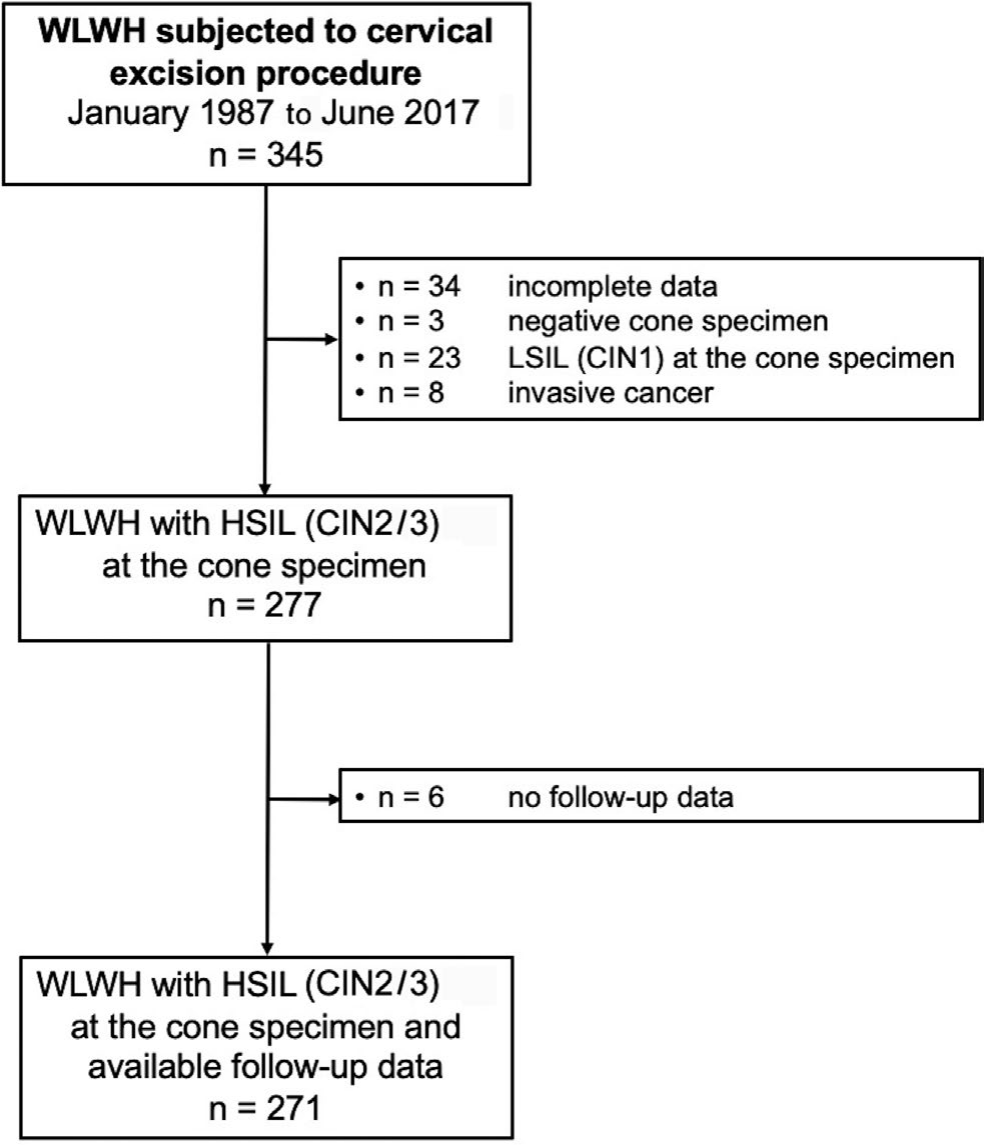
Flowchart of study population

Among the 277 WLWH diagnosed with HSIL (CIN2/3) at the cone specimen, six patients with no documented follow up were excluded, leaving 271 patients diagnosed with HSIL (CIN2/3) for the analysis of disease recurrence. Figure 1 presents the flowchart of the study population.

The median (IQR) duration of follow up was 44 (17–88) months. Among the 271 women retained in follow up, 213 (78.6%) women had no sign of recurrent disease. A recurrence of HSIL+ was found in 58 (21.4%) women. The median (IQR) time to recurrence was 18 (7–43) months. Among those patients, there were six cases (2.2%) of invasive cancer diagnosed: two at 12 months, two at 24–36 months, and two more than 60 months after treatment. The overall rate of subsequent invasive cervical cancer was 2.2% (6/271 patients). All cases of recurrence underwent a second excisional procedure.

The Kaplan‐Meier analysis at 180 months of follow up showed a probability of negative follow up at 24 months of 85.5% (standard error [SE] 2.0%), at 60 months of 78.3% (SE 2.9%), at 120 months of 72.3% (SE 3.6%), and at 180 months of 63.9% (SE 5.2%).

We evaluated the influence of all sociodemographic, clinical, and HIV‐specific variables as risk factors for disease recurrence with the log‐rank test (Table 1). Two factors were associated with the recurrence of high‐grade disease with a *P* value <0.05: age 41 years or more and HR‐HPV positivity at follow up; those factors were considered as covariates in the subsequent Cox proportional hazards regression model. Both included factors that were significantly and independently associated with a higher risk of disease recurrence: age 41 years or more with a relative risk (RR) of 4.15 (95% confidence interval [CI] 2.01–8.58, *P* < 0.001) and HR‐HPV positivity during follow up with an RR of 5.18 (95% CI 2.12–12.67, *P* < 0.01).

**TABLE 1:** Logrank test for factors associated with cumulative disease recurrence at 180 months in 271 women with diagnosis of CIN2/3 at the cone specimen^a^

Factor	Population (*n* = 271)	Hazard ratio (95% CI) for disease recurrence	*P* value
Age, years			
≤29	71 (26.2%)	1.29 (0.71–2.35)	0.373
30–35	81 (29.9%)	0.56 (0.33–0.97)	0.062
36–40	56 (20.7%)	0.57 (0.31–1.03)	0.107
≥41 years	63 (23.2%)	2.34 (1.18–4.62)	0.001
First sexual intercourse ≤16 years	133 (49.1%)	0.63 (0.38–1.08)	0.087
No. of partners five or fewer	125 (46.1%)	0.74 (0.44–1.24)	0.262
Smoker	185 (68.3%)	1.05 (0.60–1.82)	0.866
Nulliparous	135 (49.8%)	0.91 (0.54–1.51)	0.702
Menopause	13 (4.8%)	1.69 (0.47–6.08)	0.303
No ART at inclusion	49 (18.1%)	0.64 (0.33–1.25)	0.259
Pre‐HAART at inclusion	79 (29.2%)	1.23 (0.70–2.16)	0.444
HAART at inclusion	143 (52.7%)	1.04 (0.62–1.75)	0.870
WHO Class			
“Severe” at inclusion	62 (22.9%)	1.24 (0.65–2.34)	0.486
“Advanced” at inclusion	57 (21.0%)	1.46 (0.77–2.75)	0.194
“Mild” at inclusion	77 (28.4%)	0.74 (0.42–1.29)	0.309
“Not significant” at inclusion	75 (27.7%)	0.80 (0.45–1.41)	0.450
HR‐HPV positivity at inclusion^b^	113/126 (89.7%)	0.65 (0.16–2.71)	0.471
LEEP	221 (81.6%)	0.88 (0.45–1.70)	0.681
Laser	35 (12.9%)	0.94 (0.44–2.04)	0.880
Cold knife	15 (5.5%)	1.51 (0.51–4.51)	0.371
Positive endocervical margin	36 (13.3%)	1.47 (0.65–3.33)	0.282
No ART at follow up	30 (11.1%)	0.68 (0.29–1.60)	0.442
Pre‐HAART at follow up	48 (17.7%)	1.28 (0.64–2.54)	0.446
HAART at follow up	193 (71.2%)	1.05 (0.59–1.87)	0.875
WHO Class			
“Severe” at follow up	62 (22.9%)	1.49 (0.79–2.82)	0.170
“Advanced” at follow up	42 (15.5%)	1.05 (0.51–2.17)	0.884
“Mild” at follow up	49 (18.1%)	0.93 (0.46–1.85)	0.829
“Not significant” at follow up	118 (43.5%)	0.76 (0.45–1.27)	0.293
HR‐HPV positivity at follow up^b^	89/148 (60.1%)	4.03 (2.07–7.85)	<0.001

Abbreviations: ART, antiretroviral therapy; CIN, cervical intraepithelial neoplasia; HAART, highly active antiretroviral therapy; HR, high‐risk; LEEP, loop electrical excision procedure.

^a^ Values are given as number (percentage) and hazard ratio (95% confidence interval).

^b^ High‐risk‐HPV was performed in 126 women at inclusion.

^c^ High‐risk‐HPV was performed in 148 women at follow up.

**FIGURE 2:**
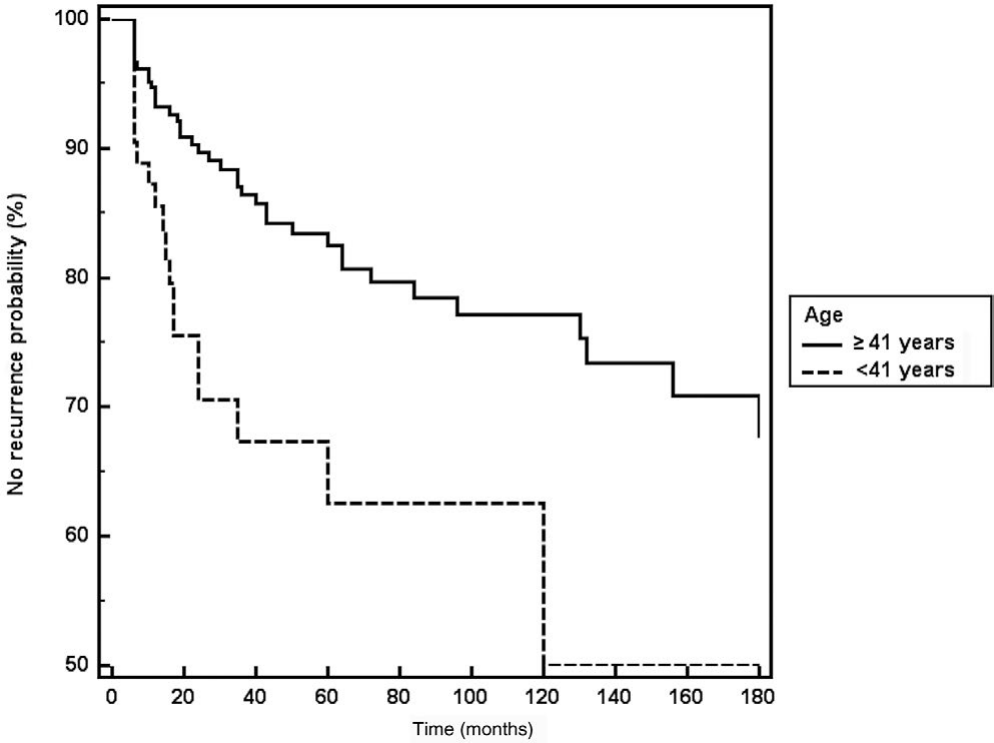
Kaplan‐Meier curve for disease recurrence according to age 41 years or more or less than 41 years at the time of treatment

**FIGURE 3:**
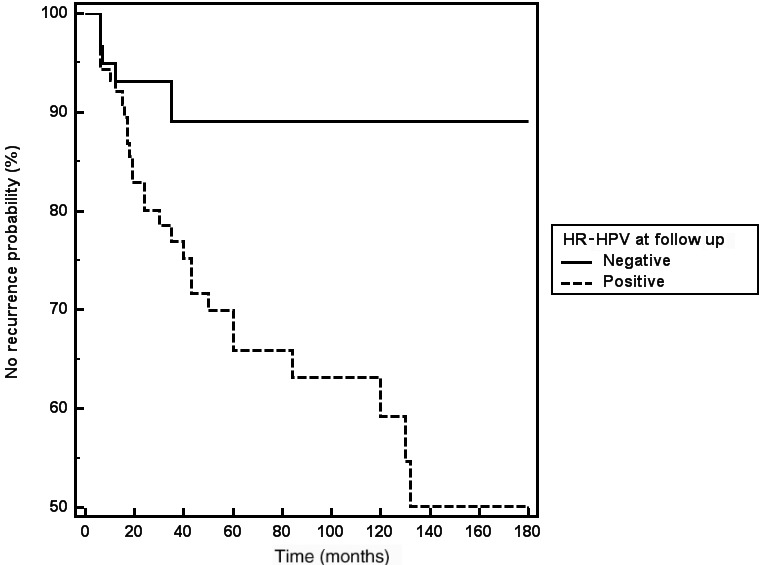
Kaplan‐Meier curve for disease recurrence according to high‐risk‐HPV positivity during follow up

Figure 2 reports the Kaplan‐Meier survival curves with respect to age 41 years or more, and Figure 3 the Kaplan‐Meier survival curves according to HR‐HPV positivity during follow up.

The HR‐HPV positivity showed a sensitivity of 82.9% (95% CI 66.4%–93.4%), a specificity of 46.9% (95% CI 37.5%–56.5%), a positive predictive value of 32.6% (95% CI 27.8%–37.8%), and a negative predictive value of 89.8% (95% CI 80.6%–94.9%) with regard to disease recurrence.

The log‐rank test of risk factors for HR‐HPV positivity during follow up is reported in Table 2. Age between 30 and 35 years and a WHO Class “Not significant” at follow up were associated with a lower risk of HR‐HPV positivity, whereas age of 41 years or older, smoking, a “Severe” WHO Class at inclusion, and a WHO Class “Severe” at follow up were associated with a higher risk of HR‐HPV positivity during follow up. All these factors were included in a Cox proportional‐hazards regression model. Only one factor related to a higher risk of HR‐HPV positivity during follow up was retained in the model: age 41 years or more, with an RR of 1.75 (95% CI 1.01–3.04, *P *= 0.047).

**TABLE 2:** Logrank test for factors associated with HR‐HPV positivity during follow up in 271 women with diagnosis of CIN2/3 at the cone specimen^a^

Factor	Population (*n* = 271)	Hazard ratio (95% CI) for HR‐HPV positivity during follow up	*P* value
Age, years			
≤29	71 (26.2%)	1.48 (0.91–2.42)	0.073
30–35	81 (29.9%)	0.51 (0.33–0.79)	0.005
36–40	56 (20.7%)	0.90 (0.55–1.46)	0.658
≥41	63 (23.2%)	1.69 (0.95–3.00)	0.028
First sexual intercourse ≤16 years	133 (49.1%)	1.32 (0.87–2.00)	0.180
No. of partners five or fewer	125 (46.1%)	0.79 (0.52–1.20)	0.254
Smoker	185 (68.3%)	1.64 (1.05–2.55)	0.043
Nulliparous	135 (49.8%)	1.15 (0.76–1.74)	0.509
Menopause	13 (4.8%)	1.31 (0.42–4.08)	0.589
No ART at inclusion	49 (18.1%)	1.14 (0.64–2.03)	0.642
Pre‐HAART at inclusion	79 (29.2%)	0.67 (0.44–1.03)	0.068
HAART at inclusion	143 (52.7%)	1.33 (0.88–2.02)	0.159
WHO Class			
“Severe” at inclusion	62 (22.9%)	1.86 (1.04–3.33)	0.008
“Advanced” at inclusion	57 (21.0%)	1.16 (0.71–1.88)	0.534
“Mild” at inclusion	77 (28.4%)	0.80 (0.51–1.24)	0.320
“Not significant” at inclusion	75 (27.7%)	0.66 (0.42–1.05)	0.100
HR‐HPV positivity at inclusion	113/126 (89.7%)	0.47 (0.09–2.44)	0.183
LEEP	221 (81.6%)	0.97 (0.44–2.12)	0.935
Laser	35 (12.9%)	1.34 (0.14–12.96)	0.769
Cold knife	15 (5.5%)	0.99 (0.43–2.26)	0.984
Positive endocervical margin	36 (13.3%)	1.05 (0.52–2.12)	0.890
No ART at follow up	30 (11.1%)	0.81 (0.40–1.65)	0.582
Pre‐HAART at follow up	48 (17.7%)	1.43 (0.75–2.73)	0.208
HAART at follow up	193 (71.2%)	0.87 (0.52–1.44)	0.549
WHO Class			
“Severe” at follow up	62 (22.9%)	1.72 (0.98–3.02)	0.021
“Advanced” at follow up	42 (15.5%)	1.07 (0.61–1.89)	0.801
“Mild” at follow up	49 (18.1%)	1.28 (0.66–2.49)	0.417
“Not significant” at follow up	118 (43.5%)	0.61 (0.40–0.93)	0.017

Abbreviations: ART, antiretroviral therapy; CIN, cervical intraepithelial neoplasia; HAART, highly active antiretroviral therapy; HR, high‐risk; LEEP, loop electrical excision procedure.

^a^ Values are given as number (percentage) and hazard ratio (95% confidence interval).

^b^ HR‐HPV was performed in 126 women at inclusion.

^c^ HR‐HPV was performed in 148 women at follow up.

## 4 DISCUSSION

The results from the present study showed that cervical excision is an effective procedure for high‐grade CIN treatment in WLWH, considering that 78.6% of women presented a long‐term negative follow up. However, the risk of recurrent high‐grade lesion or invasive cancer seems to last for many years. In our cohort, women aged 41 years or more had a four times higher risk of recurrence of HSIL+, and women with HR‐HPV positivity during follow up had a five times higher risk. Moreover, age 41 years or more was found to be a risk factor for HR‐HPV positivity during follow up.

The 21.4% HSIL+recurrence rate found in the present study is in agreement with those reported for WLWH, summarized in the recent meta‐analysis from Debeaudrap et al.,^7^ which reported a range from 15.8% to 27.0%. However, comparison of our results with those of previous studies is difficult because persistent and recurrent lesions have often been considered together, and the severity of CIN has not always been classified.

High‐risk HPV positivity during follow up is a recognized risk factor for both short‐term and long‐term recurrence of cervical lesions after treatment in HIV‐negative women.^11^ This association has been evaluated less frequently in WLWH, and data are not as consistent. Indeed, whereas Massad et al. ^8^ reported a hazard ratio of 2.9 in case of HPV positivity for recurrence of any grade, Lodi et al. ^18^ affirmed that high‐risk HPV subtypes were detected in most cases but were not associated with recurrence.

Immunosuppression, expressed as low CD4^+^ T‐cell count, is considered one of the most significant risk factors for disease recurrence in WLWH.^3^, ^19^

In our study, CD4^+^ T‐cell count at the time of cervical treatment or at the time of the last control was not associated with a higher risk of recurrence. Literature data about the most appropriate prognostic CD4^+^ T‐cell count are conflicting. Clifford et al.^19^ showed that nadir CD4^+^ T‐cell count was a more discriminant measure of risk for CIN2+ than CD4^+^ T‐cell count at diagnosis. Other studies found that CD4^+^ T‐cell count at the time of treatment is a better predictor of persistent disease or recurrence than nadir CD4^+^ T‐cell count.^8^ Our results could be interpreted as the fact that the pathogenesis of high‐grade cervical lesions is multifactorial, and immunodeficiency could play a role as a cofactor, but not as a determining factor. Previous studies reporting this association may have been biased by not having evaluated HR‐HPV positivity during follow up as a risk factor for recurrence. The role of immunodeficiency in disease relapse could be a factor increasing the risk of acquiring and maintaining HR‐HPV infection rather than in a direct role in the development of a new lesion. This finding is consistent with the existing literature suggesting that alterations in cell‐mediated immune responses play a large role in the persistence of HPV infection.^20^

The use of HAART allows for prolonged suppression of HIV replication and improved immune status, as manifested in rising CD4 cell counts. It seems to be associated with a reduction of the incidence, progression, and recurrence of cervical lesions, especially if started at higher CD4^+^ T‐cell count and used over longer durations by adherent patients.^21^ It could be speculated that HAART could be implicated in the effectiveness of the immune response in eliminating HPV infection immediately after the cervical treatment, preventing the persistence of subclinical infection and, therefore, the finding of an HR‐HPV positivity during follow up. This effect could be in some way similar to the hypothesized mechanisms by which HPV vaccination before treatment could boost the immune response, keeping the virus under control and preventing it from reactivating. However, these mechanisms are still uncertain, and future studies are needed in this regard.^22^

In the WLWH included in our cohort, HR‐HPV test during follow up after cervical excisional treatment presented a high negative predictive value (89.8%, 95% CI 80.6%–94.9%), allowing reassurance to negative patients about the low risk of recurrence, but the expected low positive predictive value (32.6%, 95% CI 27.8%–37.8%) implies the need for execution of further diagnostic tests (cervical cytology and colposcopy) to exclude disease recurrence.

Being HPV negative appears to be the most important protective factor against disease recurrence. HPV vaccination could therefore represent an additional protective factor after cervical excision, allowing a higher percentage of women to maintain the status of HPV negativity. HPV vaccination could also prevent new HPV infections, which in a long‐term follow up could be confusing regarding the true incidence of high‐grade disease persistence of new‐onset cervical lesions. Future studies are undoubtedly needed regarding HPV vaccination after treatment in WLWH; at present, there are already ongoing study protocols for HPV vaccination after treatment in HIV‐negative women.^23^ If this approach proves effective, it could be evaluated with further studies also in WLWH.

The increased risk of high‐grade recurrence for patients aged 41 years or older that emerged from our data could be explained by the age‐related immunosenescence of older WLWH that is reported to occur earlier than in HIV‐negative women of the same age.^24^ Moreover, age has been reported as a risk factor for recurrence after cervical excision also in HIV‐negative women, although it may be of less importance than other factors.^25^ In our study, we did not find any statistically significant association with sociodemographic characteristics (except age), procedural modalities, or margin involvement. It is interesting to note that smoking was not a risk factor for disease recurrence in our cohort, unlike what is reported in the literature.^21^, ^22^ This lack of association could be related to the high percentage of smokers in our population (68.3%).

As also reported in a recent meta‐analysis,^11^ HPV positivity during follow up seems to play a more important role than the positive margins in predicting disease recurrence. This may be even more relevant in a cohort of immunocompromised patients like WLHW, such as those included in our study.

The major strengths of our study are the large cohort of WLWH subjected to cervical treatment considered for the analysis, the extensive look at clinical data and pre/postoperative variables, the long‐term follow up (up to 5 years from treatment for most patients), and its multicentric nature. Moreover, the outcome chosen in the study for recurrence (HSIL+) might have reduced the probability of detecting transient low‐grade lesions. In addition, the long period of time covered by our study also allowed us to evaluate the potential effect of changes in ART, up to the current therapeutic regimens, that can provide longer lives for patients and reduce HIV transmission. The successes of ART have reduced HIV to a chronic condition in many parts of the world as progression to AIDS has become rare.^26^

Limitations of our study include its retrospective nature, the absence of HPV genotyping, and the fact that HR‐HPV testing was not performed in all the included women because it only entered clinical practice from 2002. In addition, it was not possible to discriminate if high‐grade recurrence occurring many years after treatment could be the result of a new HR‐HPV infection. However, the presented data about long‐term follow up are necessary to deepen the history of these patients. Moreover, it also needs to be acknowledged that CD4^+^ count at the time of treatment may not have the same clinical significance for each included WLWH, and generalization from these data may be limited from the heterogeneous immunologic management of the included women because of temporal changes in HAART use.

In conclusion, surgical excision of CIN seems to be an effective procedure in WLWH, with a high rate of negative follow up. However, a careful, intensive, and prolonged follow up after treatment is necessary. As the main risk factor for long‐term recurrence seems to be HR‐HPV positivity during follow up, the implementation of this test is needed in WLWH with a history of cervical excisional treatment with more systematic use of co‐testing.

In our opinion, the interval of follow up after treatment could be individualized according to the patient's age. Whereas in young women it is possible to adopt a follow‐up interval similar to that used in HIV‐negative women, older women should be followed at yearly intervals after 24 months from treatment.

## CONFLICTS OF INTEREST

The authors have no conflicts of interest.

## AUTHOR CONTRIBUTIONS

AA and CA contributed to the conception of the work; DCG, SF, GL, GB, SM, FMG, and PA contributed to the design of the work; DCG, SM, MM, DFA, FMM, and MC contributed to acquisition and analysis of data; AA, CA, DCG, and SF contributed to interpretation of data; DCG, SM, and GL drafted the work; AA and CA revised it critically for important intellectual content; all authors approved the final version to be published; and all authors agreed to be accountable for all aspects of the work in ensuring that questions related to the accuracy or integrity of any part of the work are appropriately investigated and resolved.
